# Development and psychometric properties of a questionnaire to assess barriers to feeding critically ill patients

**DOI:** 10.1186/1748-5908-8-140

**Published:** 2013-12-04

**Authors:** Naomi E Cahill, Andrew G Day, Deborah Cook, Daren K Heyland

**Affiliations:** 1 Department of Public Health Sciences, Queen’s University, Carruthers Hall, Kingston, Ontario, Canada; 2 Department of Medicine, Queen’s University, Etherington Hall, Kingston, Ontario, Canada; 3 Clinical Evaluation Research Unit, Kingston General Hospital, Stuart Street, Kingston, Canada; 4 Departments of Medicine, Clinical Epidemiology & Biostatistics, McMaster University, Main Street West, Hamilton, Ontario, Canada

**Keywords:** Barriers, Critical care, Factor analysis, Guideline implementation, Instrument development, Nutrition, Reliability, Validity

## Abstract

**Background:**

To successfully implement the recommendations of critical care nutrition guidelines, one potential approach is to identify barriers to providing optimal enteral nutrition (EN) in the intensive care unit (ICU), and then address these barriers systematically. Therefore, the purpose of this study was to develop a questionnaire to assess barriers to enterally feeding critically ill patients and to conduct preliminary validity testing of the new instrument.

**Methods:**

The content of the questionnaire was guided by a published conceptual framework, literature review, and consultation with experts. The questionnaire was pre-tested on a convenience sample of 32 critical care practitioners, and then field tested with 186 critical care providers working at 5 hospitals in North America. The revised questionnaire was pilot tested at another ICU (n = 43). Finally, the questionnaire was distributed to a random sample of ICU nurses twice, two weeks apart, to determine test retest reliability (n = 17). Descriptive statistics, exploratory factor analysis, Cronbach alpha, intraclass correlations (ICC), and kappa coefficients were conducted to assess validity and reliability.

**Results:**

We developed a questionnaire with 26 potential barriers to delivery of EN asking respondents to rate their importance as barriers in their ICU. Face and content validity of the questionnaire was established through literature review and expert input. The factor analysis indicated a five-factor solution and accounted for 72% of the variance in barriers: guideline recommendations and implementation strategies, delivery of EN to the patient, critical care provider attitudes and behavior, dietitian support, and ICU resources. Overall, the indices of internal reliability for the derived factor subscales and the overall instrument were acceptable (subscale Cronbach alphas range 0.84 – 0.89). However, the test retest reliability was variable and below acceptable thresholds for the majority of items (ICC’s range −0.13 to 0.70). The within group agreement, an indices reflecting the reliability of aggregating individual responses to the ICU level was also variable (ICC’s range 0.0 to 0.82).

**Conclusions:**

We developed a questionnaire to identify barriers to enteral feeding in critically ill patients. Additional studies are planned to further revise and evaluate the reliability and validity of the instrument.

## Background

Clinical practice guidelines (CPGs) focusing on nutrition therapy for mechanically ventilated critically ill patients have been developed to assist practitioners in the intensive care unit (ICU) manage the rapid proliferation of new information in this area, and make informed feeding decisions [1-5]. However, despite the publication and dissemination of these CPGs, there is considerable variation in nutrition practice across ICUs. Large gaps exist between many recommendations and observed practice [6-10]. Consequently, on average, the delivery of nutrition is suboptimal, with patients only receiving 59% of the calories that they are prescribed [10]. Adopting the practices recommended by these guidelines is associated with significant reductions in length of stay, infectious complications, and mortality [11-13]. Consequently, efforts to implement guideline recommendations and narrow this gap in quality care are warranted [14].

Understanding the barriers to change is key to optimal healthcare delivery [15]. Tailoring guideline implementation interventions to address identified barriers to nutrition guideline implementation may be a more effective strategy than the ‘one size fits all’ approach adopted previously in this area [16-19]. However, to identify barriers to change, valid, reliable assessment methods are needed [20].

Barriers may be identified using quantitative and qualitative methods, including observation, focus group discussions, interviews, surveys of providers, or through analysis of the organization or system. Each method has strengths and weaknesses, although surveys have the advantage of enabling data collection on a large representative sample of providers and tracking change across time. A recent systematic review of 256 studies evaluating barriers to guideline adherence [15], observed that most of these studies (n = 178) used a questionnaire to identify barriers. However, the survey-type instruments adopted in these studies were not rigorously designed. While several questionnaires have undergone some psychometric testing [21-24], they were primarily developed for specific guidelines (*e.g.*, hand hygiene), certain professional groups (*e.g.*, nurses), or unique clinical contexts (*e.g.*, primary care). Therefore, the suitability of these questionnaires for administration to multidisciplinary critical care providers is uncertain. Furthermore, it has been proposed that to be useful for selecting tailored interventions, barriers need to be measured specific to the type of innovation and local context [25].

The objective of this report is to describe the development and psychometric evaluation of a questionnaire designed to assess barriers to adherence to critical care nutrition guidelines for enterally feeding critically ill patients.

## Methods

### Conceptual framework

Cabana *et al.* reviewed 76 studies that assessed the potential barriers to physician adherence to CPGs and assimilated the results into a framework [26]. We selected this framework to guide our research because it aligned with our specific objective of identifying barriers to guideline adherence. We adopted a case study approach [27] to revise and extend this framework and make it more applied to barriers to adherence to nutrition guidelines in the ICU and to include both individual provider level and system level barriers [28]. Several other authors have also revised and expanded this framework to make it more applicable for specific guidelines or innovations [15,29-31].

The multiple case study was conducted in four ICUs in Canada between February and April 2006 [32], it included semi-structured interviews with 28 critical care providers (*i.e.*, physicians, nurses, and dietitians) to ascertain attitudes and perceptions about nutrition guidelines. The qualitative analysis of the interview transcripts, related ICU documents, and field notes was guided by Cabana *et al.*’s framework [26]. Textual coding was conducted independently by two researchers, and relationships between these codes were identified to create key thematic domains, which resulted in a framework for barriers to adherence to critical care nutrition guidelines. The schema and explanatory tables that describe this framework have been published elsewhere [28], but briefly the five thematic domains and associated sub-categories included in the framework were: guideline characteristics; implementation process; institutional factors (*i.e.*, hospital and ICU structure, hospital processes, resources, ICU culture); provider intended behavior (*i.e.*, provider characteristics (*i.e.*, professional role, critical care expertise, educational background, personality), knowledge (*i.e.*, familiarity, awareness), attitudes (*i.e.*, agreement, outcome expectancy, motivation, self-efficacy); and patient characteristics. Table 1 illustrates the framework domains, potential barriers to adherence of critical care nutrition guidelines, and example of potential questionnaire items. Patient preferences, lack of reimbursement, and malpractice liability were barriers present in Cabana *et al.*’s framework that were not observed to be relevant to critical care nutrition guidelines. The strategy or process for implementing the guideline, the prevailing culture of the ICU [33], and the characteristics of the ICU provider were new themes included in the revised framework. In addition, the term provider intent was chosen to replace physician behavior to better reflect the interdisciplinary nature of critical care, and since an individual’s intention to follow a guideline may not be synonymous with actual behavior [34,35].

**Table 1:** Framework for adherence to critical care nutrition clinical practice guidelines

**Thematic domain and sub-domain**	**Barrier**	**Example of potential Item**
**CPG Characteristics**	● Outdated	Current scientific evidence supporting some nutrition interventions is inadequate to inform practice.
	● Vague or complex statements	
	● Lack of evidence	
***Implementation Process***	● Lack of availability of all ICU Team to attend meetings, educational sessions etc.	Not enough time dedicated to education and training on how to optimally feed patients.
	● No dedicated individual willing to ‘champion’ the guidelines	
	● Time commitment to develop and implement educational strategies	
	● Restricted access to computers	
	● Displacement of posters and pamphlets over time	
**Institutional Characteristics**		
Hospital and ICU Structure	● Community hospital	N/A (*i.e.*, non actionable barriers)
	● Open structure	
	● Rural location	
	● Small hospital and/or ICU	
	● Lack of geographical consolidation	
Hospital Processes	● Long, slow administrative process	Our ICU Managers/Directors are [not] supportive of implementing nutrition guidelines.
	● Disconnect between priorities of management and clinical personnel	
	● Organizational constraints on practice	
Resources for Implementation	● Shortage of staff	Not enough nursing staff to deliver adequate nutrition.
	● Limited budget	
	● Lack of appropriate equipment/materials	
	● Lack of access to specialist services	
*Prevailing Culture of ICU*	● No cohesive, multi-disciplinary team structure	Our ICU team [does not] engage in joint decision-making in planning, coordinating and implementing nutrition therapy for our patients.
	● No multi-disciplinary daily rounds	
	● Unresolved conflict or disagreements between ICU team members	
	● Reliance on written communication (*e.g.*, Cardex, paper notes)	
	● Leadership not physically present on unit	
	● Poor communication	
***Provider Intent to Adhere***		
*Provider Characteristics*		
*Professional Roles*	● Circle of influence of nursing staff and allied healthcare professionals (*e.g.*, dietitian) dependent on support of physician and leadership team	I [do not] feel responsible for ensuring that my patients receive adequate nutrition while in the ICU.
*Critical Care Expertise*	● Junior, novice staff	
	● Locum or casual staff	
*Educational Background*	● Clinical training >10 years	
	● Reliance on expert opinion	
*Personality*	● Type B personality (i.e. relaxed and easygoing)	
	● Uncooperative	
	● Laggard/skeptic	
Knowledge		
Familiarity	● CPGs infrequently used due to rare clinical condition or narrow case-mix	I am not familiar with our current guidelines for nutrition in the ICU.
Awareness	● Conflicting and numerous CPGs on same topic	There is not enough time dedicated to education and training on how to optimally feed patients.
	● Information overload	
	● Time required to remain updated	
	● Poor dissemination	
Attitudes		
Outcome Expectancy	● Experience of adverse event from following guideline	Fear of adverse events due to aggressively feeding patients.
		General belief among ICU team that provision of adequate nutrition does not impact on patient outcome.
Self-efficacy (*i.e.*, belief that one does not have the capability to perform the actions required to implement the recommendation [36])	● Labour-intensive	My lack of skills on how to achieve goal calories.
	● Complex procedure	
	● Limited circle of influence	
Motivation	● Inertia of previous practice, especially among experienced, older staff	I am [not] willing to change my routines and habits in order to implement the recommendations of nutrition guidelines.
	● Resistance to change, especially locums, surgeons and non-ICU physicians.	
	● High cost/work burden associated with following the guideline	
Agreement	● Paucity of evidence supporting recommendation	Current scientific evidence
	● Lack of generalizability to critical care and/or specific patient groups	supporting some nutrition interventions is inadequate to inform practice.
**Patient Characteristics**	● Poor prognosis	In resuscitated, hemodynamically stable patients, other aspects of patient care still take priority over nutrition.
	● Other priorities of care	
	● Unstable clinical condition or contraindication	
	● Surgical patients	
	● Reconciliation with family preferences	

*Italics* = new themes/sub-categories not included in Cabana *et al.*’*s* knowledge-attitudes-behavior framework [26].

Individual interview summaries and the framework were sent to each key informant who participated in the case study for review and feedback. In addition, we held two face-to-face meetings with experts in the content area (*i.e.*, ICU physicians and providers specializing in nutrition—*e.g.*, dietitians, nurses, and physicians—respectively), and presented our findings to them to assess the comprehensiveness, clarity, and face validity of the developed framework.

### Item generation

The purpose of the questionnaire was to assess barriers to be targeted for change through a tailored guideline implementation strategy [19]. To this end, we intended the questionnaire to be administered to individual providers to determine their perception of the barriers to enterally feeding patients in the ICU in which they primarily work. To maximize the usefulness of the questionnaire, *a priori* it was decided to focus only on barriers that are amenable to change and can be targeted by intervention strategies to improve practices, rather than non-actionable barriers (*e.g.*, patient case-mix). Acknowledging that national or society guidelines are frequently adapted locally, the questionnaire did not refer to any specific set of published ICU nutrition guidelines but asked respondents to refer to the guidelines currently being used to inform decisions about feeding in their respective ICUs. In addition, we focused on recommendations related to enteral nutrition (EN) only, rather than parenteral nutrition, nutrient supplementation, or nutritional assessment, because these recommendations are uniformly endorsed across published guidelines [1-5], are supported by the highest level of evidence, and ICU providers generally agree with the recommendations [37].

In addition to our conceptual framework [28], potential items were identified through a literature review of studies of barriers to guideline adherence and by examining the content of existing barrier questionnaires developed in other settings [21-24]. This initial list of potential items was circulated to experts to obtain input on item comprehensiveness and wording. Redundant or irrelevant items (*i.e.*, represented non-modifiable barriers, or were not applicable to the ICU) were eliminated. Following item generation and reduction, in December 2009, a draft paper-based version of the questionnaire composed of 62 items, including 53 potential barriers, divided into four sections was pre-tested with a convenience sample of 32 critical care practitioners (11 physicians, 11 nurses, and 10 dietitians) from across Canada. Based on this pre-test, the questionnaire was revised and reduced further to 49 items, including 39 potential barriers, divided into four parts (Additional file 1).

Part A consisted of general questions about the ICU environment and the implementation of guidelines (nine items). Part B asked respondents about their level of agreement with the recommendations of critical care nutrition CPGs pertaining to enteral feeding (eight items). Part C focused on barriers to delivering adequate amounts of EN (22 items). Each item in Part A, B, and C used a seven-point Likert scale, to maximize the potential to discriminate among barriers and to allow a neutral response [35]. The items in Part A and B were formulated positively and end-anchored by the adjectives ‘1 = fully disagree’ and ‘7 = fully agree’ and included a ‘don’t know’ option. Parts A and B were intended to assess attitudes towards nutrition in general and the guideline recommendations specifically, because attitudes may influence an individual’s intention to feed and subsequent behavior, such that lack of agreement with these items indicates a barrier to feeding critically ill patients. The items in Part C were formulated negatively and end-anchored with the adjectives ‘1 = not at all important’ and ‘7 = very important’, with ‘very important’ indicating that the item is a major barrier and ‘not important’ indicating that it is not a barrier in their ICU. Each item in Part A, B, and C maps on to one of the five domains of the framework. In addition, Part C included four open-ended questions asking respondents to list additional important barriers to delivering adequate EN in their ICU, to list the most important barriers in their ICU, and to highlight strategies to overcome these barriers. In Part D (six items), characteristics of the respondent are captured.

### Field test

The sampling pool for field testing was provided by seven ICUs from five hospitals in North America who were participating in a pre-test post-test study evaluating the feasibility of a tailored guideline implementation strategy (The PERFECTIS study [ClinicalTrials.gov identifier: NCT01168128]). At each ICU, in March 2010, all full and part-time physicians, nurses, dietitian(s), the Nurse Manager, and the ICU Manager were invited to complete the questionnaire (n = 409). If the nursing pool exceeded 85, a random sample of 60 nurses was used. To maximize the response rate, the questionnaire was distributed according to a modified Dillman’s tailored design method [38], and respondents were provided with the option of completing a web-based (survey monkey [39]), electronic (fillable pdf), or paper-based version of the questionnaire.

### On-site observational visits

To confirm the results of the field testing and further refine the questionnaire, in May and June 2010 we conducted on-site observational visits at all five hospitals in the field test. Half-day focus groups were completed with ICU physician and nursing leaders, bedside nurses, and dietitian(s). Participants were first asked to reflect on EN provision in their ICU and identify areas where they perform well and areas for improvement. During these discussions, we explored the reasons (*i.e.*, barriers and enablers) for high or poor performance. Attendees were asked to rank the identified barriers in order of their negative impact on the provision of nutrition (*i.e.*, considering the degree of delay it caused and the frequency of its occurrence). Results of the barriers questionnaire were then presented to the group and the top ten ranked barriers from the questionnaire were compared with the rankings provided by attendees in the earlier discussion. A detailed report of these on-site visits is published elsewhere [40].

### Data analysis to determining the psychometric properties of the questionnaire

First, we conducted a descriptive analysis (*e.g.*, missing data, variance, mean, histograms etc.). The frequency of non-response was examined, and items with a non-response of greater than 10% were reviewed and considered for re-wording or eliminated. The standard frequency distributions of responses to each item in the questionnaire were then examined for floor and ceiling effects. Items with a very high (>0.8) or low (<0.2) endorsement frequency (*i.e.*, proportion of respondents responded ‘fully agree’, ‘agree’, or ‘somewhat agree’ in Part A or B and ‘’very important’, ‘important’, or ‘somewhat important’ in Part C) were considered for elimination, because responses to these items can be predicted and including them does not improve the scales psychometric properties [41].

### Exploratory factor analysis

To refine the content of the barriers questionnaire, reduce the number of items and ensure the most parsimonious representation of the underlying constructs, we conducted an exploratory factor analysis.

Missing values were treated as truly missing without imputation. A principal components analysis with varimax (orthogonal) rotation and kaiser normalization was used [42]. Eigenvalues of >1 (Kaiser criteria), the cumulative percentage of variance explained by successive factors, a scree plot, and at least three items with factor loadings greater than 0.5 were used, together with the underlying conceptual framework, to identify the number of factors [42]. Factor loadings of >0.5 were considered acceptable for item retention on a single factor [42]. Items that cross-loaded at >0.5 or loaded 0.4 – 0.5 on a single factor were evaluated by the research team on a case-by-case basis, retained or eliminated based on the item’s conceptual importance, its unique contribution to the factor, and whether it was strongly related conceptually to another factor. Following the descriptive and exploratory factor analysis, we revised the questionnaire.

### Internal reliability

We examined the reliability of the overall questionnaire and evaluated factor analysis derived subscales using Cronbach’s alpha. As individual questionnaire responses were intended to be aggregated to the ICU level to identify barriers pertinent to the ICU and inform a tailored intervention, a minimum overall and subscale co-efficient alpha of 0.8 was considered desirable, and any item for which alpha significantly increased if the item was deleted from the scale was considered for removal [41].

### Scoring the questionnaire

As the purpose of the questionnaire was to identify barriers to target through a tailored intervention, when scoring the questionnaire we focused on the upper end of the seven-point Likert scale. Item scores were calculated by awarding 1, 2, or 3 points if the respondent identified an item as a ‘somewhat important’, ‘important’ or ‘very important’ barrier respectively. If an item was rated 1 – 4 (*i.e.*, ‘not at all important’ to ‘neither important or unimportant’ it was awarded 0 points. The barrier score was calculated by dividing the awarded points for each item by the maximum number of potential points (*i.e.*, 3), and multiplied by 100. Each factor identified by the exploratory factor analysis was considered as a subscale, so that subscale and overall barriers scores were calculated as the mean score of all items within that subscale and mean of all items respectively. Subsequent analyses were conducted using the barriers score.

### Aggregating responses to the unit level

To be a useful tool for tailoring interventions it was also important to assess the extent to which individual responses approximate the barriers within their ICU. To this end, respondents were instructed to complete the questionnaire so that responses reflected the average situation in their ICU. To assess whether responses might be aggregated to the ICU level to obtain a single estimate of site-level barriers, we used three indices of within-group agreement and group mean reliability to examine each questionnaire item, subscale, and overall score [43]: intraclass correlation coefficient (1) (ICC [1]) (or Shrout and Fleiss model 1,1 [44]); ICC(2) (or Shrout and Fleiss model 1,k where k = 35 respondents [44]); and the F-test p-value. The variance components to compute the ICCs were calculated using mixed linear regression models with Restricted Maximum Likelihood (REML) estimation, and the F-test p-values were derived from a one-way analysis of variance (ANOVA). The aggregated data were considered reliable if the F tests’ p-values were <0.05 indicating that responses differ in different ICUs and/or ICC(2) (an estimate of the reliability of group means) was >0.60 [45]. ICC(1) is the ratio of between-group variance to total variance and is an estimate of the degree of reliability associated with a single providers assessment of the unit mean. Values of ICC(1) between 0.05 and 0.20 are typical in organizations [43].

### Qualitative analysis

Responses to the open-ended questions were reviewed to determine whether respondents identified barriers that were not already included in Part C.

We reviewed minutes from the focus group sessions at the five field test sites for evidence supporting content and construct validity (*i.e.*, to identify additional barriers, and evaluate if themes emerging from the focus groups mapped on to the identified factor structure).

### Pilot testing

Following completion of the analysis of the field test data, the research team met to review the results and revise the questionnaire. A revised version of the barriers questionnaire was circulated to ICU providers who had provided feedback on earlier drafts during the pre-test and field test. In March 2011, the final version of the barriers questionnaire was pilot-tested in a simple random sample of 60 providers working in a 20 bed closed ICU at a 404 bed teaching hospital in Canada. Using an open-ended format, respondents were asked for feedback and to report the time to completion; we made further revisions where required.

### Test-retest

Finally, in May 2011 we administered the barriers questionnaire to a simple random sample of 60 full- and part-time nurses working in a 16 bed closed ICU in a 472 bed Canadian teaching hospital to assess test-retest reliability. The questionnaire was distributed on two occasions, two weeks apart, using the same methods of distribution as in the field test. ICC (Shrout and Fleiss model 2,1 [44]) was calculated between the original item, mean subscale and mean overall responses at the two time points. An ICC >0.7 was considered acceptable [46]. For each item, we also dichotomized nurses responses based on their rating of importance (*i.e.*, ≤4 = not a barrier and >4 = barrier) and calculated kappa co-efficients. A kappa of 0.0 – 0.2 was considered poor agreement, 0.2 – 0.4 as fair agreement, 0.4 – 0.6 as moderate agreement, 0.6 – 0.8 as substantial agreement, and 0.8 – 1.0 as perfect agreement [47]. To further assess the degree of agreement and to identify potential bias Bland and Altman plots were also produced [48].

### Sample size

*A priori*, we estimated that each ICU participating in the field test would have approximately 80 staff members to whom the questionnaire would be distributed, and that the response rate would be approximately 50%, giving a sample size of 280 and a sample size to item ratio of 7 to 1. This sample size surpasses the recommended minimum of 150 cases and a sample size to number of items ratio of no lower than 4 to 1 for exploratory factor analysis [49]. For assessment of test retest reliability, we aimed to distribute 60 – 85 questionnaires, giving a sample of 30 – 43 when accounting for the anticipated response rate of 50%. This exceeds to 15 – 20 subjects recommended for estimating reliability [50].

### Ethical considerations

The Queen’s University Health Sciences and Affiliated Teaching Hospitals Research Ethics Board, Kingston, Ontario and the seven hospitals participating in the field, pilot, and reliability testing approved this study (REB# EPID-292-09 and DMED-994-06). Return of the completed questionnaire and/or attendance at the focus groups implied informed consent on the part of participating critical care providers.

## Results

### Field test

#### Descriptive statistics

A total of 186 completed questionnaires out of 409 distributed questionnaires (45.5%) were received. Tables 2 and 3 describe the characteristics of the five participating hospitals and the field test respondent demographics, respectively.

**Table 2:** Characteristics of and response rate at the five hospitals participating in the field test

**ICU**^**#**^	**Country**	**Hospital type**	**Hospital size**	**ICU structure**	**ICU size**	**Response rate n/N (%)**
1	USA	Non-Teaching	361	Closed^&^	20	37/73 (50.7)
2	Canada	Teaching	497	Closed	16	32/85 (37.7)
3*	USA	Teaching	600	Open^	32	36/98 (36.7)
4	Canada	Non-Teaching	400	Open	13	29/73 (39.7)
5	Canada	Teaching	759	Closed	30	52/80 (65.0)

*Three units combined due to common infrastructure and shared staffing.

^#^ICU = Intensive Care Unit.

^Open = patient under care of any attending physician.

^&^Closed = patient under care of an intensivist.

**Table 3:** Personal characteristics of field test sample

**Characteristic**	**N (%)**
Sex	N = 171
Male	28 (16.4)
Female	143 (83.6)
Age	N = 172
20 – 34	75 (43.6)
35 – 49	68 (39.5)
≥ 50	29 (16.9)
Clinical Specialty	N = 186
Dietitian	25 (13.4)
Nurse	138 (74.2)
Physician	12 (6.5)
Other^	11 (5.9)
Time dedicated to ICU^#^	N = 173
Full-time	120 (69.4)
Part-time	45 (26.0)
Other^&^	8 (4.6)
Length of time working in critical care	N = 173
0 – 5	77 (44.5)
6 – 10	45 (26.0)
11 – 15	20 (11.6)
>15	31 (17.9)
Leadership role*	N = 171
Yes	53 (31.0)
No	118 (69.0)

*Examples of a leadership role include charge nurse, clinical nurse specialist, nurse manager ^#^ICU = Intensive Care Unit.

^*e.g.*, pharmacist, nurse attendant, student nurse, resident.

^&^*e.g.*, casual, trainee placement.

Descriptive statistics of the individual questionnaire items are shown in Table 4. *A priori*, we planned to eliminate items with a high proportion of missing data or high endorsement frequency; a commonly applied strategy in initial item reduction [49]. In our field test, the proportion of missing values did not exceed 10% for any item, but endorsement frequency was high for the majority of items in Parts A and B. Greater than 80% of respondents agreed with the majority of statements in these sections, resulting in medians skewed to the left and little variance in responses (see Table 4). The endorsement frequency was less that 80% for only 2 items in Part A (A.8 with 67% and A.9 with 77%) and two in Part B (B.4 with 61% and B.7 with 78%). The two items in Part A were retained and reworded to become negative statements (*i.e.*, ‘I am not familiar with our current guidelines for nutrition in the ICU’ and ‘General belief among ICU team that provision of adequate nutrition does not impact on patient outcome.’ During the on-site focus groups it was highlighted that at some sites the thresholds recommended by these two guideline recommendations in Part B may differ from the local policy documents with which they are familiar (*e.g.*, local policy might tolerate a gastric residual volume of 200 ml not >250 ml as stated in the guidelines or may advocate raising the head of bed to 30 degrees and not 45 degrees as recommended by the guidelines). Based on these observations, we surmised that if the thresholds used in the questionnaire items corresponded to the local thresholds, endorsement would have exceeded 80%, and therefore these items were eliminated.

**Table 4:** Descriptive statistics of barrier questionnaire items

**Framework domain**	**Item**	**Median**	**Interquartile range**	**Mode**	**Range**	**Don’t know (%)**	**Missing (%)**	**Endorsement (%)**
**Part A: General barriers**								
Institutional Characteristics	*1. Overall, our unit functions very well together as a team.*	6.0	6 – 7	6.0	3 – 7	1 (0.5)	1 (0.5)	96.2
Institutional Characteristics	*2. Our ICU team engages in joint decision-making in planning, coordinating and implementing nutrition therapy for our patients.*	6.0	6 – 7	6.0	1 – 7	1 (0.5)	0	93.6
Institutional Characteristics	*3. Overall, it is easy for me to openly talk with other members of the ICU team about matters related to the nutritional needs of my patient.*	7.0	6 – 7	7.0	1 – 7	1 (0.5)	0	95.7
Institutional Characteristics	*4. In our ICU, implementing best practices, as defined by clinical practice guidelines, is intrinsic to our culture.*	6.0	6 – 7	6.0	2 – 7	2 (1.1)	1 (0.5)	93.0
Institutional Characteristics	*5. Our ICU Managers/Directors are supportive of implementing nutrition guidelines.*	6.0	6 – 7	7.0	2 – 7	7 (3.8)	1 (0.5)	82.7
Provider Intent	*6.* *Nutrition is very important for my critically ill patients.*	7.0	7 – 7	7.0	6 – 7	2 (1.1)	1 (0.5)	98.9
Provider Intent	*7. I feel responsible for ensuring that my patients receive adequate nutrition while in the ICU.*	7.0	6 – 7	7.0	5 – 7	1 (0.5)	3 (1.6)	99.5
Provider Intent	***8. I am familiar with our current national guidelines for nutrition in the ICU.***	6.5	4 – 6	6.0	2 – 7	10 (5.4)	0 (0.0)	67.2
Provider Intent	***9. If the recommendations of the current national guidelines for nutrition are followed in our ICU, patient outcomes will improve.***	6.0	5 – 7	6.0	1 – 7	19 (10.2)	0 (0.0)	77.4
**Part B. Guideline recommendations for enteral nutrition**								
Provider Intent	*1. Enteral nutrition should be used in preference to parenteral nutrition.*	7.0	6 – 7	7.0	4 – 7	3 (1.6)	0 (0.0)	95.7
Provider Intent	*2. Enteral nutrition should be initiated early (24 – 48 hours following admission to ICU).*	6.0	6 – 7	7.0	3 – 7	1 (0.5)	0 (0.0)	97.9
Provider Intent	*3. An evidence-based feeding protocol should be used.*	7.0	6 – 7	7.0	2 – 7	1 (0.5)	1 (0.5)	96.8
Provider Intent	*4. If a feeding protocol is used, it should tolerate a higher gastric residual volume (i.e., >250 mls) before holding feeds.*	6.0	3 – 7	7.0	1 – 7	9 (4.8)	1 (0.5)	60.5
Provider Intent	*5. In patients who have feed intolerance (i.e., high gastric residual volumes, emesis) a promotility agent should be used.*	6.0	6 – 7	6.0	1 – 7	3 (1.6)	0 (0.0)	96.2
Provider Intent	*6. Small bowel feeding should be considered for those select patients who repeatedly demonstrate high gastric residual volumes and are not tolerating adequate amounts of enteral nutrition delivered into the stomach.*	6.0	6 – 7	6.0	1 – 7	10 (5.4)	0 (0.0)	91.4
Provider Intent	*7. Patients receiving enteral nutrition should have the head of the bed elevated to 45 degrees.*	6.0	5 – 7	7.0	1 – 7	2 (1.1)	2 (1.1)	78.3
Provider Intent	*8. In all critically ill patients, hyperglycemia (blood glucose >10 mmol/l or 180mg/dl) should be avoided by minimizing intravenous dextrose and using insulin administration when necessary.*	6.0	6-7	6.0	2-7	2 (1.1)	0 (0.0)	94.6
**Part C: Barriers to the provision of enteral nutrition in the Intensive Care Unit**								
Institutional characteristics	1. Not enough nursing staff to deliver adequate nutrition.	3.0	2 – 5	1.0	1 – 7		2 (2.2)	30.2
Institutional characteristics	2. Not enough dietitian time dedicated to the ICU during regular weekday hours.	3.0	2 – 6	2.0	1 – 7		5 (2.7)	38.1
Institutional characteristics	3. No or not enough dietitian coverage during weekends and holidays.	5.0	3 – 6	6.0	1 – 7		5 (2.7)	60.8
Institutional characteristics	4. Enteral formula not available on the unit.	4.0	2 – 6	6.0	1 – 7		4 (2.2)	50.0
Institutional characteristics	5. No or not enough feeding pumps on the unit.	5.0	2 – 6	6.0	1 – 7		5 (2.7)	58.0
Guideline characteristics	6. Current scientific evidence supporting some nutrition interventions is inadequate to inform practice.	4.0	2 – 5	5.0	1 – 7		13 (7.0)	46.8
Guideline characteristics	7. The current national guidelines for nutrition are not readily accessible when I want to refer to them.	5.0	2 – 6	5.0	1 – 7		7 (3.8)	55.3
Guideline characteristics	8. The language of the recommendations of the current national guidelines for nutrition are not easy to understand.	4.0	2 – 5	4.0	1 – 7		11 (5.9)	38.3
Implementation Process	9. Not enough time dedicated to education and training on how to optimally feed patients.	5.0	3 – 6	5.0	1 – 7		6 (3.2)	57.8
Implementation Process	10. No feeding protocol in place to guide the initiation and progression of enteral nutrition.	4.0	2 – 5	1.0	1 – 7		7 (3.8)	45.3
Implementation Process	11. Current feeding protocol is outdated.	4.0	2 – 5	4.0	1 – 7		13 (7.0)	34.1
Provider intent	12. Delay in physicians ordering the initiation of EN.	5.0	3 – 6	5.0	1 – 7		5 (2.7)	65.2
Provider intent	13. Waiting for the dietitian to assess the patient.	4.0	2 – 6	6.0	1 – 7		6 (3.2)	48.3
Provider intent	14. Non-ICU physicians (*i.e.*, surgeons, gastroenterologists) requesting patients not be fed enterally.	5.0	3 – 6	6.0	1 – 7		6 (3.2)	57.8
Provider intent	15. Nurses failing to progress feeds as per the feeding protocol.	4.0	2 – 6	6.0	1 – 7		4 (2.2)	45.6
Provider Intent	16. Fear of adverse events due to aggressively feeding patients.	4.0	2 – 5	5.0	1 – 7		5 (2.7)	48.6
Provider Intent	17. Feeding being held too far in advance of procedures or operating room visits.	5.0	2 – 6	5.0	1 – 7		6 (3.2)	58.9
Provider Factor	18. No feeding tube in place to start feeding.	5.0	2 – 6	6.0	1 – 7		4 (2.2)	54.4
Patients Factor	19. Delays in initiating motility agents in patients not tolerating enteral nutrition (*i.e.*, high gastric residual volumes).	5.0	3 – 6	5.0	1 – 7		4 (2.2)	55.5
Patient Factor	20. Delays and difficulties in obtaining small bowel access in patients not tolerating enteral nutrition (*i.e.*, high gastric residual volumes).	5.0	4 – 6	6.0	1 – 7		5 (2.7)	67.4
Patient Factor	21. In resuscitated, hemodynamically stable patients, other aspects of patient care still take priority over nutrition.	5.0	4 – 6	6.0	1 – 7		5 (2.7)	68.0
Institutional characteristic/Patient Factor	***22. Lack of agreement among ICU team on the best nutrition plan of care for the *** **patient.**	3.0	2 – 5	2.0	1 – 7		4 (2.2)	32.4

Framework domain column indicates which of the five thematic domains of our previously developed framework [28] that the specific questionnaire item maps on to.

Responses options for Part A: 1 = fully disagree 2 = disagree 3 = somewhat disagree 4 = no opinion 5 = somewhat agree 6 = agree 7 = fully agree Endorsement = % respondents who responded ‘fully agree,’ ‘agree’ or ‘somewhat agree’ in Part A.

Responses options for Part B: 1 = fully disagree 2 = disagree 3 = somewhat disagree 4 = no opinion 5 = somewhat agree 6 = agree 7 = fully agree.

Endorsement = % respondents who responded ‘fully agree,’ ‘agree’ or ‘somewhat agree’ in Part B.

Response options for Part C: 1 = not at all important 2 = unimportant 3 = somewhat unimportant 4 = neither important or unimportant 5 = somewhat important 6 = important 7 = very important. Endorsement = % respondents responded ’very important,’ ‘important’ or ‘somewhat important’ in Part C. Items ordered in table as per questionnaire distributed during field test.

*Italics =* items eliminated due to high endorsement frequency ***bolded italics*** *=* items retained but reworded.

For the 22 items in Part C, greater variance was observed for the majority of items. As a consequence of the overall high endorsement frequency Parts A and B (focused on general barriers and agreement with recommendations respectively), were omitted from subsequent psychometric assessment.

### Factor analysis

Observations with missing values on any of the items in Part C were omitted from the factor analysis (27 of 186 observations) resulting in 159 respondents for this analysis. The principal components analysis indicated a five-factor solution accounting for 72% of the variance. The eigenvalues for each factor and the factor loadings for each item after orthogonal rotation are shown in Table 5. The identified factors closely reflected the conceptual framework that guided the development of the barriers questionnaire.

**Table 5:** Barriers questionnaire factor analysis and internal reliability

**Item number**	**Questionnaire items and new subscale names**	**Eigenvalue**	**Variance explained**	**Loading on Factor 1**	**Loading on Factor 2**	**Loading on Factor 3**	**Loading on Factor 4**	**Loading on Factor 5**	**Alpha**	**Alpha if item deleted**
	**Subscale 1: Guideline Recommendations and Implementation Strategies**	10.01	47.67						0.89	
C6	● Current scientific evidence supporting some nutrition interventions is inadequate to inform practice.			**0.68**	0.15	0.30	0.24	0.23		0.87
C7	● The current guidelines for nutrition are not readily accessible when I want to refer to them.			**0.84**	0.19	0.07	0.17	0.20		0.86
C8	● The language of the recommendations of the current guidelines for nutrition are not easy to understand.			**0.77**	0.25	0.12	0.12	0.31		0.85
C10	● No feeding protocol in place to guide the initiation and progression of enteral nutrition.			**0.54**	0.38	0.15	0.34	0.31		0.87
C11	Current feeding protocol is outdated.			**0.63**	0.31	0.21	0.31	0.14		0.86
	**Subscale 2: Delivery of Enteral Nutrition to the Patient**	1.68	8.00						0.86	
C12	● Delay in physicians ordering the initiation of EN.			0.19	**0.42**	0.41	0.45	0.17		0.85
C18	No feeding tube in place to start feeding.			0.26	**0.82**	0.12	0.12	0.27		0.81
C19	● Delays in initiating motility agents in patients not tolerating enteral nutrition (*i.e.*, high gastric residual volumes).			0.19	**0.78**	0.32	0.07	0.24		0.81
C20	● Delays and difficulties in obtaining small bowel access in patients not tolerating enteral nutrition (*i.e.*, high gastric residual volumes).			0.16	**0.72**	0.27	0.27	−0.02		0.84
C21	● In resuscitated, hemodynamically stable patients, other aspects of patient care still take priority over nutrition.			0.32	**0.52**	0.17	0.23	0.17		0.85
	**Subscale 3: Critical Care Provider Attitudes and Behavior**	1.20	5.72						0.87	
C14	● Non-ICU physicians (*i.e.*, surgeons, gastroenterologists) requesting patients not be fed enterally.			−0.24	0.27	**0.67**	0.31	0.04		0.83
C15	● Nurses failing to progress feeds as per the feeding protocol.			0.09	0.26	**0.82**	0.09	0.19		0.79
C16	● Fear of adverse events due to aggressively feeding patients.			0.33	0.24	**0.60**	0.07	0.33		0.84
C17	● Feeding being held too far in advance of procedures or operating room visits.			0.10	0.11	**0.87**	0.15	0.07		0.81
	**Subscale 4: Dietitian Support**	1.13	5.36						0.84	
C13	● Waiting for the dietitian to assess the patient.			0.37	0.26	0.19	**0.63**	0.18		0.79
C2	● Not enough dietitian time dedicated to the ICU during regular weekday hours.			0.03	0.26	0.09	**0.70**	0.49		0.80
C3	● No or not enough dietitian coverage during evenings, weekends and holidays.			0.27	0.13	0.15	**0.77**	0.19		0.77
C9	● There is not enough time dedicated to education and training on how to optimally feed patients.			0.51	0.08	0.29	**0.60**	−0.05		0.83
	**Subscale 5: ICU Resources**	1.10	5.23						0.84	
C1	● Not enough nursing staff to deliver adequate nutrition.			0.15	0.25	0.22	0.38	**0.66**		0.84
C4	● Enteral formula not available on the unit.			0.31	0.23	0.07	0.24	**0.74**		0.71
C5	● No or not enough feeding pumps on the unit.			0.32	0.08	0.21	0.04	**0.80**		0.75
	**Eliminated/Reworded Item**									
C22	● Lack of agreement among ICU team on the best nutrition plan of care for the patient.			0.23	0.46	0.25	0.42	0.35		

Bolded text = items retained in the factor.

#### Factor one: guideline recommendations and implementation strategies

‘Guideline recommendations’ and ‘Guideline implementation strategies’ were identified as two separate domains in the conceptual framework. However, in our factor analysis, the first factor included all six items from these two domains, although item C.9 loaded more heavily on factor three.

#### Factor two: delivery of EN to the patient

Items associated with the ‘Patient factor’ domain of the conceptual framework were represented in the second factor. Item C.22 did not load on any factor at the cut-off criteria of 0.5 but did load at 0.458 on factor two. However, following feedback at the focus group sessions and discussion among the investigative team, the item was reworded to better reflect the barrier of poor communication rather than lack of agreement (*i.e.*, ‘Needles delays in relaying information regarding the initiation and progression of nutrition’). Item C.22 was therefore omitted from subsequent analyses. *A priori,* we hypothesized that item C.12 (*i.e.*, Delay in physician ordering the initiation of EN) would be associated with the ‘critical care provider intent domain.’ In our factor analysis, it did not load on any factor at the cut-off of >0.5 but loaded on factors two, three, and four at 0.42, 0.40, and 0.45 respectively. Despite loading more highly on factor four (Dietitian support), we considered this to be more theoretically aligned with the items in factor two. The title of this factor was changed from ‘Patient factor’ to ‘Delivery of EN to the patient’ to better reflect specific barriers that lead to a delay in EN provision.

#### Factor three: critical care provider attitudes and behavior

The third factor represented the items associated with the ‘Critical care provider intent’ domain of the framework. Two items (C.12 and C.13) originally conceptualized to belong to this domain loaded on other factors. The investigative team agreed that the remaining items reflected behaviors that arose from attitudinal beliefs about nutrition and how best to feed ICU patients; therefore the title of this factor was changed to ‘Critical care provider attitudes and behavior’ to better reflect this association.

#### Factor four: dietitian support

Four items referring to the role of the dietitian (C2, C3, C9, C13), identified in numerous domains of the original conceptual framework, were represented by a single factor in the analysis.

#### Factor five: ICU resources

The fifth factor represented the items associated with the ‘ICU environment’ domain of the original framework. The title of this factor was changed to ‘ICU resources’ to better reflect that the factor focused on the barrier of inadequate staff and equipment rather than the general environment.

### Internal reliability

The Cronbach alpha coefficient for the barriers scale was 0.94. The alpha coefficients for the factor subscales all exceeded the acceptable cut-off of >0.8, ranging from 0.84 to 0.89.The alpha when an item was deleted remained stable for each item, with the exception of one item in factor five (Not enough nursing staff to deliver adequate nutrition) (Refer to Table 5).

### Aggregating responses to the unit level

The variance components and indices to assess the reliability of aggregating individual provider responses to the unit level are shown in Table 6. A total of 11 of the 21 questionnaire items included in the analysis and subscales two, three, four, and 5, demonstrated statistically significant F statistics and ICC(1,35) values >0.6 in the acceptable range. The ICC (1,1) was greater than 0.05 typically observed in the organizational literature for 10 of the 21 items. However, the values for all three indices were not acceptable for the overall and subscale one scores.

**Table 6:** Statistical justification for aggregating data to the unit level

	**Site specific barrier score mean ± SD**					**Variance components**				**F-test P-value**
**Questionnaire items**	**Site 1**	**Site 2**	**Site 3**	**Site 4**	**Site 5**	**σ**_**b**_^2*^	**σ**_**w**_^**2#**^	**ICC^**	**ICC (35)**^**&**^	
**N**	**37**	**32**	**36**	**29**	**52**					
**Overall Barriers**	**32.2 ± 26.9**	**31.3 ± 20.9**	**33.3 ± 33.3**	**39.9 ± 35.5**	**26.8 ± 19.0**	**7.67**	**522.66**	**0.01**	**0.34**	**0.2**
**Subscale 1: Guideline Recommendations and Implementation Strategies**	**27.5 ± 28.6**	**38.2 ± 25.0**	**22.4 ± 27.9**	**26.4 ± 21.6**	**21.4 ± 21.0**	**0.00**	**602.27**	**0.00**	**0.00**	**0.77**
Current scientific evidence supporting some nutrition interventions is inadequate to inform practice.	23.8 ± 31.9	28.7 ± 34.2	18.6 ± 29.8	29.5 ± 30.3	22.4 ± 26.7	0.00	908.80	0.00	0.00	0.6
The language of the recommendations of the current guidelines for nutrition are not easy to understand.	28.7 ± 33.9	26.4 ± 34.9	18.6 ± 29.8	33.3 ± 33.3	12.2 ± 27.8	46.18	998.05	0.04	0.62	0.03
The current guidelines for nutrition are not readily accessible when I want to refer to them.	35.2 ± 35.6	31.1 ± 31.5	24.5 ± 35.1	34.6 ± 35.2	33.3 ± 35.5	0.00	1196.27	0.00	0.00	0.71
No feeding protocol in place to guide the initiation and progression of enteral nutrition.	25.0 ± 33.2	25.3 ± 34.1	31.4 ± 33.3	26.2 ± 29.2	19.6 ± 28.4	0.00	984.31	0.00	0.00	0.56
Current feeding protocol is outdated.	23.8 ± 36.7	16.0 ± 29.8	19.0 ± 30.6	15.4 ± 25.4	20.7 ± 26.8	0.00	891.70	0.00	0.00	0.8
**Subscale 2: Delivery of Enteral Nutrition to the Patient**	**33.0 ± 31.2**	**38.2 ± 25.0**	**39.3 ± 27.0**	**54.6 ± 29.0**	**30.5 ± 22.5**	**62.18**	**714.14**	**0.08**	**0.75**	**0.005**
Delay in physicians ordering the initiation of EN.	33.3 ± 39.0	41.1 ± 28.6	43.5 ± 36.4	49.4 ± 39.6	30.1 ± 29.0	29.36	1175.27	0.02	0.47	0.11
No feeding tube in place to start feeding.	31.5 ± 39.0	24.4 ± 34.9	37.0 ± 33.6	59.5 ± 34.4	26.3 ± 31.9	155.32	1197.87	0.11	0.82	0.0006
Delays in initiating motility agents in patients not tolerating enteral nutrition (*i.e.*, high gastric residual volumes).	25.9 ± 37.5	35.6 ± 37.1	33.3 ± 30.9	53.6 ± 36.7	24.4 ± 28.1	97.41	1124.24	0.08	0.75	0.004
Delays and difficulties in obtaining small bowel access in patients not tolerating enteral nutrition (*i.e.*, high gastric residual volumes).	34.3 ± 37.8	48.9 ± 32.4	40.7 ± 34.8	56.8 ± 33.1	32.1 ± 31.6	70.09	1150.70	0.06	0.68	0.02
In resuscitated, hemodynamically stable patients, other aspects of patient care still take priority over nutrition.	39.8 ± 35.5	41.1 ± 33.5	41.7 ± 34.2	53.6 ± 37.8	37.3 ± 35.1	0.00	1236.48	0.00	0.00	0.39
**Subscale 3: Critical Care Provider Attitudes and Behavior**	**27.9 ± 30.3**	**44.7 ± 29.5**	**20.8 ± 26.2**	**31.8 ± 29.5**	**33.0 ± 24.3**	**51.15**	**764.14**	**0.06**	**0.70**	**0.01**
Non-ICU physicians (*i.e.*, surgeons, gastroenterologists) requesting patients not be fed enterally.	34.4 ± 33.3	60.0 ± 32.0	24.1 ± 33.4	30.9 ± 33.2	32.7 ± 36.2	148.80	1154.98	0.11	0.82	0.0006
Nurses failing to progress feeds as per the feeding protocol.	22,2 ± 34.7	35.6 ± 38.1	14.8 ± 25.8	27.4 ± 31.5	34.0 ± 35.2	44.12	1115.92	0.04	0.58	0.05
Fear of adverse events due to aggressively feeding patients.	28.7 ± 33.9	28.9 ± 34.7	23.1 ± 31.7	36.9 ± 36.7	24.2 ± 30.6	0.00	1096.86	0.00	0.00	0.48
Feeding being held too far in advance of procedures or operating room visits.	24.8 ± 34.6	54.4 ± 38.6	21.3 ± 31.7	33.3 ± 36.3	41.8 ± 33.2	143.22	1166.23	0.11	0.81	0.0006
**Subscale 4: Dietitian Support**	**33.3 ± 37.1**	**37.9 ± 28.0**	**28.1 ± 28.3**	**40.4 ± 25.7**	**23.4 ± 21.7**	**35.26**	**710.07**	**0.05**	**0.63**	**0.03**
Waiting for the dietitian to assess the patient.	27.6 ± 40.8	34.4 ± 33.3	27.8 ± 33.3	37.0 ± 33.8	21.8 ± 28.7	7.73	1137.44	0.01	0.19	0.32
Not enough dietitian time dedicated to the ICU during regular weekday hours.	21.3 ± 33.0	34.4 ± 33.3	21.9 ± 33.3	35.7 ± 38.4	11.5 ± 24.6	76.09	1014.70	0.07	0.72	0.005
No or not enough dietitian coverage during evenings, weekends and holidays.	30.6 ± 38.5	52.2 ± 37.8	29.5 ± 35.9	51.2 ± 34.5	32.1 ± 30.9	91.75	1241.46	0.07	0.72	0.009
There is not enough time dedicated to education and training on how to optimally feed patients.	27.8 ± 29.3	32.2 ± 35.1	31.5 ± 31.8	36.9 ± 35.5	28.1 ± 30.1	0.00	1011.24	0.00	0.00	0.78
**Subscale 5: ICU Resources**	**42.9 ± 32.8**	**28.7 ± 29.5**	**23.1 ± 30.7**	**43.7 ± 34.3**	**20.5 ± 24.4**	**95.09**	**892.58**	**0.10**	**0.79**	**0.0009**
Not enough nursing staff to deliver adequate nutrition.	18.5 ± 29.2	17.8 ± 27.3	17.6 ± 34.3	38.1 ± 42.3	10.3 ± 23.4	76.36	953.62	0.07	0.74	0.006
Enteral formula not available on the unit.	53.7 ± 44.6	33.3 ± 37.1	32.4 ± 36.1	42.9 ± 38.3	17.9 ± 28.4	149.67	1330.97	0.10	0.80	0.0003
No or not enough feeding pumps on the unit.	56.5 ± 42.0	37.9 ± 38.5	19.4 ± 32.2	50.0 ± 38.0	33.3 ± 33.7	172.74	1343.58	0.11	0.82	0.0003

Variance components calculated using mixed linear regression model with Restricted Maximum Likelihood estimation (REML): *= (i.e., ICU), σ_b_^2^ = between group (*i.e.*, ICU) variance # σ_w_^2^ = within group (*i.e.*, ICU) variance ^ICC = Intraclass correlation coefficient (1,1) = σ_b_^2^ / (σ_b_^2^ + σ_w_^2^) ^&^ICC(1,35) = σ_b_^2^/( σ_b_^2^ + σ_w_^2^/k) where k = 35 respondents per group.

Bolded text = overall and subscale results.

### Open-ended questions

A total of 52 out of 186 respondents (28%) completed the open-ended question ‘are there any other barriers that hinder your ability to deliver adequate amounts of EN?’ Of these, 22 indicated ‘no’ and 22 described a barrier that was already included in the questionnaire or a non-modifiable barrier (*e.g.*, patient’s clinical condition) and therefore were not considered further. Of the remaining eight responses, four described feeds being held for diarrhea, and four described waiting for x-ray confirmation of tube placement as important barriers. The latter two barriers were highlighted for inclusion as new items in the revised questionnaire.

### On-site observational visits

A total of 46 providers participated in the five focus groups, ranging from three to 14 attendees per group. Overall, at each site the important barriers reported by the attendees during the discussions on nutrition performance were conceptually the same as the top ranked barriers derived from the responses of ICU providers at their site.

### Revised barriers questionnaire and pilot test

Of the 39 potential barriers in the field test version of the questionnaire, seven items in Part A and the eight items in Part B were omitted from the revised version, leaving 24 of the original items. Of these items, three (A.8, A.9, and C.22) were reworded. In addition, two new items were added for a total of 26 potential barriers. The revised version of the barriers questionnaire presented (Additional file 2 and available at http://www.criticalcarenutrition.com) in this paper consists of two sections. The first section lists the 26 potential barriers to delivery of EN and asks the respondent to rate their importance as barriers in their ICU (See Table 7). These items are followed by two open-ended questions. The first open-ended question asked respondents if there are any other barriers that hinder their ability to deliver adequate EN, and the second asked respondents to rank the three most important of 26 potential barriers to the provision of adequate EN in their ICU. Part B includes six questions about the personal demographics of the respondent. The revised questionnaire has 34 questions.

**Table 7:** Test retest (N = 17)

**Questionnaire items**	**ICC (2,1)**	Kappa*
**Overall Barriers**	**0.64**	**0.35**
**Guideline Recommendations and Implementation Strategies**	**0.31**	**0.06**
1. Current scientific evidence supporting some nutrition interventions is inadequate to inform practice.	0.36	0.24
2. The language of the recommendations of the current guidelines for nutrition are not easy to understand.	0.37	0.38
3. **I am not familiar with our current guidelines for nutrition in the ICU.**	0.35	0.23
4. The current guidelines for nutrition are not readily accessible when I want to refer to them.	0.51	0.30
5. No feeding protocol in place to guide the initiation and progression of enteral nutrition.	−0.13	−0.03
Current feeding protocol is outdated.	0.31	0.20
**ICU Resources**	**0.57**	**0.60**
6. Not enough nursing staff to deliver adequate nutrition.	0.70	0.60
7. Enteral formula not available on the unit.	0.34	0.27
8. No or not enough feeding pumps on the unit.	0.51	0.27
**Dietitian Support**	**0.39**	**0.34**
9. Waiting for the dietitian to assess the patient.	0.15	0.21
Not enough dietitian time dedicated to the ICU during regular weekday hours.	0.43	0.34
10. No or not enough dietitian coverage during evenings,weekends and holidays.	0.52	0.34
11. There is not enough time dedicated to education and training on how to optimally feed patients.	0.32	0.20
**Delivery of Enteral Nutrition to the Patient**	**0.55**	**0.47**
No feeding tube in place to start feeding.	0.51	0.51
12. Delay in physicians ordering the initiation of EN.	0.37	0.13
13. **Waiting for physician/radiology to read x-ray and confirm tube placement.**	0.22	0.30
14. Delays in initiating motility agents in patients not tolerating enteral nutrition (*i.e.*, high gastric residual volumes).	0.43	0.16
15. Delays and difficulties in obtaining small bowel access in patients not tolerating enteral nutrition (*i.e.*, high gastric residual volumes).	0.52	0.65
16. In resuscitated, hemodynamically stable patients, other aspects of patient care still take priority over nutrition.	0.59	0.52
17. **Needles delays in relaying information regarding the initiation and progression of nutrition.**	0.36	0.32
**Critical Care Provider Attitudes and Behavior**	**0.62**	**0.35**
18. Non-ICU physicians (*i.e.*, surgeons, gastroenterologists) requesting patients not be fed enterally.	0.57	0.43
19. Nurses failing to progress feeds as per the feeding protocol.	0.09	0.19
20. **Feeds being held due to diarrhea.**	0.46	0.50
21. Fear of adverse events due to aggressively feeding patients.	0.53	0.33
22. Feeding being held too far in advance of procedures or operating room visits.	0.69	0.65
23. **General belief among ICU team that provision of adequate nutrition does not impact on patient outcome.**	0.60	0.87

*Agreement between nurses who responded that an item was ‘somewhat important’ to ‘very important’ (5 – 7) vs ‘not at all important’ to ‘neither important or unimportant’ (1 – 4).

Items ordered in table as per questionnaire distributed during pilot test.

Bolded items = new or reworded items in the revised version of the questionnaire.

The pilot test of the revised questionnaire in a separate sample of 43 nurses (response rate 72%) demonstrated completion time was less than five minutes. No further changes were made based on the pilot test feedback.

### Test-retest

**Figure 1:**
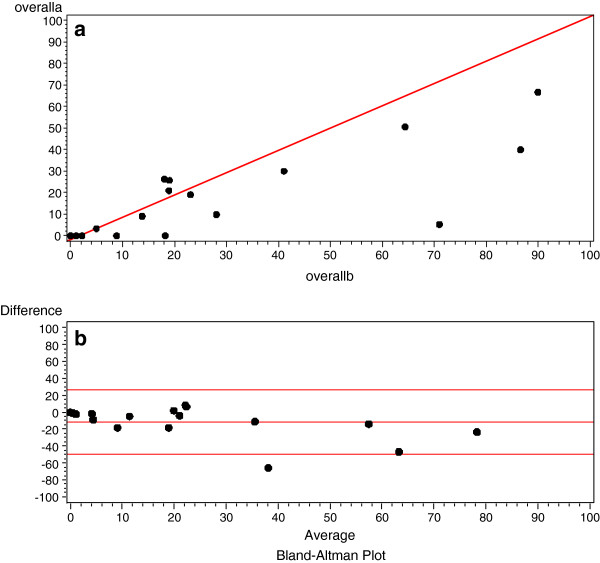
**Test retest of overall barriers score. a**. Bland Altman Line of Equality showing overall barriers score calculated from responses at time a (first administration) plotted against overall barriers score calculated at time b (two weeks later) (N = 17). **b**. Bland Altman plot showing mean overall barriers score against differences between the overall barriers score at time a (first administration) and b (two weeks later). The centre line represents zero (*i.e.*, perfect agreement). The top and bottom lines represent 95% limits of agreement (mean ± 1.96SD) so that in a randomly selected respondent from the general population the difference between the two responses would be expected to lie between these limits of agreement with approximately 95% probability (N = 17).

Of the 60 distributed questionnaires, a total of 17 nurses completed the questionnaire on two occasions, two weeks apart for a response rate of 28%. The ICC (2,1) for total barriers score was 0.64, with subscale scores ranging from 0.39 – 0.62. Only one of the individual items demonstrated acceptable correlation of >0.70. Item ICCs ranged from −0.13 to 0.70. The kappa coefficients were similar to the ICC; three items demonstrating substantial or almost perfect agreement (Table 7). The Bland Altman plots did not indicate any bias between the two observations (Figure 1a and b).

## Discussion

We aimed to develop a novel questionnaire to assess barriers to implementing guideline recommendations pertaining to enterally feeding critically ill patients and to conduct preliminary psychometric testing of this new instrument. The content of the questionnaire has a sound theoretical base, derived from a recently developed framework that describes barriers to implementation of critical care nutrition CPGs [28]. The face and content validity of the questionnaire were established through review by experts, and through pre-testing and pilot testing with ICU providers. The descriptive and exploratory factor analysis led to the elimination of several items, resulting in a more parsimonious representation of the underlying conceptual framework. The indices of internal reliability for the derived factor subscales and the overall instrument were acceptable. However, the assessment of test-retest reliability suggested that the temporal stability of the questionnaire was poor to moderate for the majority of items, with only two items demonstrating acceptable reliability.

In designing the barriers questionnaire, we hypothesized that attitudes towards nutrition and guidelines in general may function as a barrier to feeding by influencing a providers’ intent to adopt a specific recommendation and their subsequent behavior. However, our analysis revealed very high endorsement for all these general items in Parts A and B. The positive skew of the observed responses may in part be due to social desirability bias, whereby critical care providers tended to perceive their workplace favorably. However, the positive attitudes observed in our field test were also observed in our previous international survey of the attitudes of more than 500 physicians and dietitians towards the Canadian Critical Care Nutrition CPG recommendations [37]. When results of this previous survey were compared to observational data of nutrition practices [10], despite supportive evidence underlying the recommendations and uniform endorsement of these recommendations among providers, bedside practice did not follow recommendations. This supports our questionnaire field test results suggesting that negative attitudes towards guidelines and lack of agreement with ICU guideline recommendations are unlikely to be important impediments to the provision of EN.

As a first step in this program of research we developed a framework for understanding the barriers to provider adherence to critical care nutrition guidelines [28]. We used Cabana *et al*.’s knowledge-attitude-behavior framework as a starting point for our analysis [26]. Our revised framework and subsequent content of the barriers questionnaire may have differed if we had selected a different framework or theoretical model. In their qualitative study of barriers to radiography for back pain, the analysis for which was also guided by Cabana *et al*.’s framework, Espeland and Baerheim noted that this barrier classification system was similar to other systems [29]. However, different barrier systems that look at barriers from different angles may provide complementary insights [29]. Cabana *et al.* primarily used quantitative data to develop their framework; psychological theories have also been used to explain the behaviors of health professionals across different settings. Through an expert consensus process Michie *et al.* identified 12 theoretical domains of relevance to implementation research [51]. Although the terminology differs, the thematic domains and subdomains identified in our framework overlap with those in this theoretical domains framework (TDF) with the exception of our ‘patient characteristics’ domain. This suggests that the content of the barriers questionnaire may have been similar if the TDF had been used. Unlike the TDF, our intention was to use our framework and questionnaire to identify barriers that could be addressed by both behavior change and local system level change. Notwithstanding, moving forward, adopting a more theoretical approach is advised [52]. Referring to the theoretical constructs of the TDF may be useful for both informing our understanding of the nature of the identified barriers, identifying potential interventions to address them, and explaining the mechanism by which change occurs.

As the content of the barriers questionnaire was guided by the five domains of our framework for understanding adherence to guidelines in the ICU [28], we expected that the exploratory factor analysis would reveal a five-factor solution, with individual items relating to a specific domain loading onto a factor related to that domain. Although we did observe a five-factor structure to the data, there were some differences between these factors and the conceptual framework domains. The factor analysis revealed that all items relating to the dietitian’s role loaded as a distinct factor. As the dietitian has primary responsibility for nutritional care, it is intuitive that ‘dietitian support’ would be a single factor distinct from the role of other critical care providers or ICU resources. This assumption is supported by our previous analysis showing that the presence of a dietitian was associated with higher nutrition performance [53]. In contrast, items related to the two domains of guideline recommendations and guideline implementation strategies in our conceptual framework all loaded as a single factor, suggesting that ICU providers do not perceive the guideline documents and their method of implementation as different types of barriers but all related to the same concept. These observed differences with our underlying framework are not overtly discrepant but rather aid in refining it to be a more meaningful representation of potential barriers.

While preliminary evaluation revealed acceptable internal reliability, we observed that test retest reliability and the reliability of aggregated responses was poor for some items. *A priori*, we surmised that an ICU providers’ perception of barriers to enterally feeding would not change over a two-week period. Nurses may have altered their responses as a consequence of being prompted to think more about the barriers to feeding their patients following the first administration of the questionnaire, or providers may respond based on their most recent experiences with an individual patient rather than their general experience. Our sample size of 17 nurses may have been inadequate to evaluate test retest accurately. Although greater than 50% of items demonstrated acceptable reliability as aggregated variables, several items including those associated with subscale one were problematic. Items in subscale one focused on characteristics of nutrition guideline recommendations, therefore we may surmise that there will be greater variation in individuals responses surrounding these general items compared to other items focusing on routine practice in their ICU. Larger samples representing more ICUs would inform whether specific items or the response scale should be revised to improve reliability.

A weakness of the current response scale is that as the primary purpose of the scale is to identify barriers, not all the information collected on the scale is used. For example, when using the scale for the purpose of tailoring interventions or when deriving subscale and overall scores from the individual item responses we focused on the upper end of the scale only; *i.e.*, ‘5 – somewhat important', ‘6 – important’ or ‘7 – very important’ as we were not interested in factors that were not perceived to be important barriers by respondents (*i.e.*, ‘1 – not at all important’, ‘2 – unimportant’, ‘3 – somewhat important’, ‘4 – neither important or unimportant’). Consequently, by using only three points of the scale we may have lost important information regarding the magnitude of the barrier. Furthermore, these limitations associated with the response scale may have led to a reduction in the reliability of the questionnaire. Consequently, we have revised this Likert scale to better capture the degree to which each item is a barrier (*i.e.*, 0 – ‘not at all’ to 6 – ‘an extreme amount’). The usefulness and reliability of this revised scale will be assessed in future studies.

The utility of this instrument to inform quality improvement activities in the busy ICU environment is promising. In the pre-test post-test study in which this questionnaire was distributed [54], we employed multiple methods to identify barriers at participating ICUs; supplementing results of a staff survey using this questionnaire with data on the guideline-practice gap, obtained through a chart audit, and perspectives of key stakeholders, obtained through a focus group. Using the results of the barriers assessment, ICU staff prioritized barriers to target for change and selected interventions to overcome them during a one-day brainstorming session. The resulting tailored intervention was implemented over a 12-month period and we observed a statistically significant decrease in overall barriers score and a non-significant increase in prescribed calories received [54].

There are several limitations to this work. First, this report represents the first field test of the questionnaire in a convenience sample of five ICUs in North America with a moderate response rate of 46%. However, the response rate is similar to other surveys in this setting [55], and the field test sample of 159 responses used in the factor analysis provided a sample size to item ratio of 7:1 (*i.e.*, 159 responses: 22 items), surpassing the minimum recommendation of 4:1 [49]. Second, 75% of our participants were nurses; consequently the proportion of dietitians and physicians who are the primary decision makers for nutrition therapy was small. We did not involve non-ICU physicians or residents whose attitudes towards the nutrition recommendations may differ. To this end, further testing is planned in a larger international sample of providers. Third, items in the questionnaire are those that providers perceive to be important barriers to EN in the ICU, but other studies are needed to evaluate whether addressing these perceived barriers actually improves the provision of EN in practice. Finally, analyses using different datasets are required to confirm the five-factor solution derived from this field test and to establish the questionnaire’s construct validity.

## Conclusions

We have developed a questionnaire for assessing barriers to feeding critically ill patients, and have provided preliminary evidence to support the validity and internal consistency of the derived factor subscales and the overall instrument. In addition to the planned validation studies, the feasibility of using the questionnaire to identify barriers to target for change through a tailored intervention is being evaluated.

## Abbreviations

ANOVA: Analysis of variance; CPGs: Clinical practice guidelines; EN: Enteral nutrition; ICU: Intensive care unit; ICC: Intraclass correlation coefficient; REML: Restricted Maximum Likelihood.

## Competing interests

The authors declare that they have no competing interests.

## Authors’ contributions

NC and DKH were responsible for the study conception and design, obtaining the grant to fund this work, and leading the observational site visits. NC performed the data analysis and was responsible for drafting the manuscript. AD, DKH and DC provided methodological and statistical expertise, helped to interpret the results, and made critical revisions to the manuscript. All authors read and approved the final manuscript.

## Supplementary Material

Additional file 1 Barriers to Feeding Critically Ill Patients Questionnaire distributed during the field test phase of development (49 items). Click here for file

Additional file 2 Barriers to Feeding Critically Ill Patients Questionnaire revised version distributed during the pilot and test-retest phase of development (34 items). Click here for file
