# Supplementary material for: Development and psychometric properties of a questionnaire to assess barriers to feeding critically ill patients
Source: Implement Sci. 2013 Dec 4;8:140. doi: 10.1186/1748-5908-8-140 (PMC4235036; doi:10.1186/1748-5908-8-140)
Supplement: Additional file 1 — Barriers to Feeding Critically Ill Patients Questionnaire distributed during the field test phase of development (49 items). [file 1748-5908-8-140-S1.pdf]

Barriers to Feeding Critically Ill Patients
The purpose of this questionnaire is to understand the barriers that critical care providers face in implementing the recommendations of nutrition guidelines in their setting - specifically barriers to providing adequate enteral nutrition (EN).

Several Clinical Practice Guidelines (CPGs) pertaining to critical care nutrition have been developed and published in recent years (e.g., The Canadian Critical Care Nutrition Guidelines published in 2003 and updated in 2009, and the SCCM/ASPEN Guidelines for the Provision and Assessment of Nutrition Support Therapy in the Adult Critically Ill Patient published in 2009). These CPGs are typically nationally developed broad statements of best practice that refer to the average mechanically ventilated critically ill adult patient. For brevity, during this survey, the term 'current national guidelines for nutrition' will be used to refer to the nutrition guidelines currently used to inform decisions about feeding patients in your ICU.

This questionnaire is divided into 4 sections and should take you approximately 10 minutes to complete. Please read these instructions before starting:

- Read each question, including all the options, before giving an answer.
- Choose the answer that is most applicable to your situation.
- Choose only one answer, unless requested otherwise.
- Complete the questionnaire from your perspective of the situation in your ICU – do not consider what you think others would say.
- If you have any additional comments, questions or concerns regarding nutrition guidelines, barriers to delivering enteral nutrition, or this survey, please write them in the space allotted at the end of the questionnaire.

By completing the questionnaire you are consenting for your responses to be used as part of ongoing quality improvement work in your unit. However, your responses are strictly confidential. You have the option of completing the questionnaire online or completing a paper-based version of the questionnaire. Paper-based versions of the questionnaire are to be placed in the secure box provided. Questionnaires will be sent to the Clinical Evaluation Research Unit for analysis, where they will be kept in a locked office with a password-protected computer. All analyses will be based on aggregate responses only. If any single subgroup has less than 5 responses the results with be combined with another group. Your opinions are very important! Of course, your participation is voluntary.

If you have any questions or comments, please contact us:
Naomi Cahill RD MSc
Project Leader / Doctoral Candidate
Clinical Evaluation Research Unit (CERU), Angada 4,
Kingston General Hospital
Kingston, ON K7L 2V7
Tel: (613) 549 6666 X 2812
e-mail: cahilln@kgh.kari.net

Thank you for your participation!

Part A: General Barriers

Please read the following statements and circle the number that best represents your answer. By circling number 1, you are fully disagreeing with the statement, by circling number 7 you are fully agreeing with the statement.

Questions 1-5 refer to the ICU environment in which you work.

	Fully Disagree	Disagree	Somewhat disagree	No opinion	Somewhat agree	Agree	Fully agree	Don't know
1. Overall, our unit functions very well together as a team.	1	2	3	4	5	6	7	8
2. Our ICU team engages in joint decision-making in planning, coordinating and implementing nutrition therapy for our patients.	1	2	3	4	5	6	7	8
3. Overall, it is easy for me to openly talk with other members of the ICU team about matters related to the nutritional needs of my patient.	1	2	3	4	5	6	7	8
4. In our ICU, implementing best practices, as defined by clinical practice guidelines, is intrinsic to our culture.	1	2	3	4	5	6	7	8
5. Our ICU Managers/Directors are supportive of implementing nutrition guidelines.	1	2	3	4	5	6	7	8

Questions 7-9 refer to your general attitudes towards nutrition therapy and the nutrition guidelines used in your ICU.

6. Nutrition is very important for my critically ill patients.	1	2	3	4	5	6	7	8
7. I feel responsible for ensuring that my patients receive adequate nutrition while in the ICU.	1	2	3	4	5	6	7	8
8. I am familiar with our current national guidelines for nutrition in the ICU.	1	2	3	4	5	6	7	8
9. If the recommendations of the current national guidelines for nutrition are followed in our ICU, patient outcomes will improve.	1	2	3	4	5	6	7	8

Part B: Guideline Recommendations for Enteral Nutrition

This section relates to your agreement with recommendations of CPGs about enteral nutrition. Remember, these guidelines are meant to apply to the average mechanically ventilated adult critically ill patient. There are always exceptions to the rule but we are asking your level of agreement in the typical situation. Please read each statement and fill in the circle that best represents your level of agreement. By circling number 1, you are fully disagreeing with the statement, by circling number 7 you are fully agreeing with the statement.

	Fully Disagree	Disagree	Somewhat disagree	No opinion	Somewhat agree	Agree	Fully agree	Don't know
1. Enteral nutrition should be used in preference to parenteral nutrition.	**1**	**2**	**3**	**4**	**5**	**6**	**7**	**8**
2. Enteral nutrition should be initiated early (24-48 hours following admission to ICU).	**1**	**2**	**3**	**4**	**5**	**6**	**7**	**8**
3. An evidence-based feeding protocol should be used.	**1**	**2**	**3**	**4**	**5**	**6**	**7**	**8**
4. If a feeding protocol is used, it should tolerate a higher gastric residual volume (i.e. > 250mls) before holding feeds.	**1**	**2**	**3**	**4**	**5**	**6**	**7**	**8**
5. In patients who have feed intolerance (i.e. high gastric residual volumes, emesis) a promotility agent should be used.	**1**	**2**	**3**	**4**	**5**	**6**	**7**	**8**
6. Small bowel feeding should be considered for those select patients who repeatedly demonstrate high gastric residual volumes and are not tolerating adequate amounts of EN delivered into the stomach.	**1**	**2**	**3**	**4**	**5**	**6**	**7**	**8**
7. Patients receiving enteral nutrition should have the head of the bed elevated to 45 degrees.	**1**	**2**	**3**	**4**	**5**	**6**	**7**	**8**
8. In all critically ill patients, hyperglycemia (blood glucose > 10 mmol/l or 180mg/dl) should be avoided by minimizing intravenous dextrose and using insulin administration when necessary.	**1**	**2**	**3**	**4**	**5**	**6**	**7**	**8**

Part C: Barriers to Delivery of Enteral Nutrition

This section relates specifically to barriers to providing adequate enteral nutrition to patients in your ICU. A barrier is something that hinders your ability to deliver adequate amounts of EN. Below is a list of items that have been identified as barriers to feeding critically ill patients. For each potential barrier, circle the number that best reflects on average the situation in your ICU. By circling number 1 (Not at all important) you believe that it is not a barrier. By circling number 7 (Very important) you believe that it is a major barrier.

	Not at all Important	Unimportant	Somewhat Unimportant	Neither Important or Unimportant	Somewhat Important	Important	Very Important
**ICU Environment**							
1. Not enough nursing staff to deliver adequate nutrition.	1	2	3	4	5	6	7
2. Not enough dietitian time dedicated to the ICU during regular weekday hours.	1	2	3	4	5	6	7
3. No or not enough dietitian coverage during weekends and holidays.	1	2	3	4	5	6	7
4. Enteral formula not available on the unit.	1	2	3	4	5	6	7
5. No or not enough feeding pumps on the unit.	1	2	3	4	5	6	7
**Guideline Recommendations**							
6. Current scientific evidence supporting some nutrition interventions is inadequate to inform practice.	1	2	3	4	5	6	7
7. The current national guidelines for nutrition are not readily accessible when I want to refer to them.	1	2	3	4	5	6	7
8. The language of the recommendations of the current national guidelines for nutrition are not easy to understand.	1	2	3	4	5	6	7

Guideline Implementation Strategies	Not at all Important	Unimportant	Somewhat Unimportant	Neither Important or Unimportant	Somewhat Important	Important	Very Important
9. Not enough time dedicated to education and training on how to optimally feed patients.	**1**	**2**	**3**	**4**	**5**	**6**	**7**
10. No feeding protocol in place to guide the initiation and progression of enteral nutrition.	**1**	**2**	**3**	**4**	**5**	**6**	**7**
11. Current feeding protocol is outdated.	**1**	**2**	**3**	**4**	**5**	**6**	**7**

Critical Care Provider Behaviour

12. Delay in physicians ordering the initiation of EN.	**1**	**2**	**3**	**4**	**5**	**6**	**7**
13. Waiting for the dietitian to assess the patient.	**1**	**2**	**3**	**4**	**5**	**6**	**7**
14. Non-ICU physicians (i.e. surgeons, gastroenterologists) requesting patients not be fed enterally.	**1**	**2**	**3**	**4**	**5**	**6**	**7**
15. Nurses failing to progress feeds as per the feeding protocol.	**1**	**2**	**3**	**4**	**5**	**6**	**7**
16. Fear of adverse events due to aggressively feeding patients.	**1**	**2**	**3**	**4**	**5**	**6**	**7**
17. Feeding being held too far in advance of procedures or operating room visits.	**1**	**2**	**3**	**4**	**5**	**6**	**7**

Patient Factors	Not at all Important	Unimportant	Somewhat Unimportant	Neither Important or Unimportant	Somewhat Important	Important	Very Important
18. No feeding tube in place to start feeding.	**1**	**2**	**3**	**4**	**5**	**6**	**7**
19. Delays in initiating motility agents in patients not tolerating enteral nutrition (i.e. high gastric residual volumes).	**1**	**2**	**3**	**4**	**5**	**6**	**7**
20. Delays and difficulties in obtaining small bowel access in patients not tolerating enteral nutrition (i.e. high gastric residual volumes).	**1**	**2**	**3**	**4**	**5**	**6**	**7**
21. In resuscitated, hemodynamically stable patients, other aspects of patient care still take priority over nutrition.	**1**	**2**	**3**	**4**	**5**	**6**	**7**
22. Lack of agreement among ICU team on the best nutrition plan of care for the patient.	**1**	**2**	**3**	**4**	**5**	**6**	**7**

23. Reflecting on the 22 barriers to providing enteral nutrition listed above, are there any other barriers that hinder your ability to deliver adequate amounts of enteral nutrition?

24. Reflecting on the 22 barriers to providing enteral nutrition listed above, enter the number corresponding to the items that you believe are the 3 most important barriers to the provision of adequate EN in your ICU:

- First most important barrier: ☐
- Second most important barrier: ☐
- Third most important barrier: ☐

25. What strategies do you believe would improve the delivery of EN in your ICU?

26. In what format would you prefer to receive education on nutrition therapy and current nutrition guidelines? Select all that apply.

Powerpoint presentation at multidisciplinary rounds	o
Powerpoint presentation online	o
Small group in-person teaching sessions	o
One-on-one teaching sessions	o
Bed-side booklet of nutrition guideline recommendations and reference articles	o
FAQs sheet	o
Bedside illustrations (e.g. posters, head of bed elevation signs)	o
Newsletters	o
Email bulletins	o
Other, please specify: ____________________	o

<u>Part D: Personal Characteristics</u>
Please fill in the circle that best corresponds to you.

1. What is your sex? Male o Female o

2. What is your age?
   20-34 years o
   35-49 years o
   50-64 years o
   65 years or older o

3. What is your primary clinical specialty?
   Dietitian o
   Nurse o
   Physician o Please specify:
      Intensivist (Medical) o
      Intensivist (Surgical) o
      Anaesthesia o
      Emergency Medicine o
      Internal Medicine o
      Surgeon o
      Other o
   Other clinical specialty o Please specify ___________________

4. In my clinical work I am dedicated to the ICU…..
   Full-time o
   Part-time o Full-time equivalent:_______________
   Locum o
   Casual o
   Other o Please specify ___________________

5. How long have you been working in the ICU?
   0 - 5 years o
   6 - 10 years o
   11 - 15 years o
   Greater than 15 years o

6. Do you play a leadership role in the ICU? Yes o No o
   If yes, please specify:
   Medical Director o
   Nurse manager o
   Clinical Nurse Specialist o
   Charge Nurse o
   Other o

Additional Comments

You have now completed the questionnaire – thank you! In the space below, please make any additional comments you wish to make about barriers or solutions to providing adequate enteral nutrition to patients in your ICU.

Thank you very much for taking the time to complete this questionnaire. Your contribution is valued.
