# Supplementary material for: Development and psychometric properties of a questionnaire to assess barriers to feeding critically ill patients
Source: Implement Sci. 2013 Dec 4;8:140. doi: 10.1186/1748-5908-8-140 (PMC4235036; doi:10.1186/1748-5908-8-140)
Supplement: Additional file 2 — Barriers to Feeding Critically Ill Patients Questionnaire revised version distributed during the pilot and test-retest phase of development (34 items). [file 1748-5908-8-140-S2.pdf]

Critical Care Nutrition

Barriers to Feeding Critically Ill Patients

The purpose of this questionnaire is to understand the barriers that critical care providers face in implementing the recommendations of nutrition guidelines in their setting - specifically barriers to providing adequate enteral nutrition (EN).

Several Clinical Practice Guidelines (CPGs) pertaining to critical care nutrition have been developed and published in recent years. These CPGs are typically nationally developed broad statements of best practice that refer to the average mechanically ventilated critically ill adult patient. Often, these guidelines are adapted for local use, and incorporated into local policy documents and bed-side protocols/algorithms. For brevity, throughout this survey, the term 'current guidelines for nutrition' will be used to refer to the nutrition guidelines currently used, if any, to inform decisions about feeding patients in your ICU. When we refer to feeding protocols, we are referring to tools designed to operationalize the guidelines at the bedside for patient care.

This questionnaire is divided into 2 sections and should take you approximately 5 minutes to complete. Please read these instructions before starting:

- Read each question, including all the options, before giving an answer.
- Choose the answer that is most applicable to your situation.
- Choose only one answer, unless requested otherwise.
- Complete the questionnaire from your perspective of the situation in your ICU – do not consider what you think others would say.
- If you have any additional comments, questions or concerns regarding nutrition guidelines, barriers to delivering enteral nutrition, or this survey, please write them in the space allotted at the end of the questionnaire.

By completing the questionnaire you are consenting for your responses to be used as part of ongoing quality improvement work in your unit. However, your responses are strictly confidential. You have the option of completing the questionnaire online or completing a paper-based version of the questionnaire. Paper-based versions of the questionnaire are to be placed in the secure box provided in the ICU. Questionnaires will be sent to [ ] for analysis, where they will be kept in a locked office with a password-protected computer. All analyses will be based on aggregate responses only. If any single subgroup has less than 5 responses the results with be combined with another group. Your opinions are very important! Of course, your participation is voluntary.

If you have any questions or comments, please contact us:
[ ]

Thank you for your participation!

Part A: Barriers to Delivery of Enteral Nutrition

This section relates specifically to barriers to providing adequate enteral nutrition to patients in your ICU. A barrier is something that hinders your ability to deliver adequate amounts of EN. Below is a list of items that have been identified as barriers to feeding critically ill patients. For each potential barrier, circle the number that best reflects on average the situation in your ICU. By circling number 1 (Not at all important) you believe that it is not a barrier. By circling number 7 (Very important) you believe that it is a major barrier.

Not a Barrier → Major Barrier

	Not at all Important	Unimportant	Somewhat Unimportant	Neither Important or Unimportant	Somewhat Important	Important	Very Important
**Guideline Recommendations and Implementation Strategies**	**1**	**2**	**3**	**4**	**5**	**6**	**7**
1. I am not familiar with our current guidelines for nutrition in the ICU.							
2. Current scientific evidence supporting some nutrition interventions is inadequate to inform practice.	**1**	**2**	**3**	**4**	**5**	**6**	**7**
3. The language of the recommendations of the current guidelines for nutrition are not easy to understand.	**1**	**2**	**3**	**4**	**5**	**6**	**7**
4. The current guidelines for nutrition are not readily accessible when I want to refer to them.	**1**	**2**	**3**	**4**	**5**	**6**	**7**
5. No feeding protocol in place to guide the initiation and progression of enteral nutrition.	**1**	**2**	**3**	**4**	**5**	**6**	**7**
6. Current feeding protocol is outdated.	**1**	**2**	**3**	**4**	**5**	**6**	**7**
**ICU Resources**							
7. Not enough nursing staff to deliver adequate nutrition.	**1**	**2**	**3**	**4**	**5**	**6**	**7**
8. Enteral formula not available on the unit.	**1**	**2**	**3**	**4**	**5**	**6**	**7**
9. No or not enough feeding pumps on the unit.	**1**	**2**	**3**	**4**	**5**	**6**	**7**

	Not at all Important	Unimportant	Somewhat Unimportant	Neither Important or Unimportant	Somewhat Important	Important	Very Important
	Not a Barrier →						Major Barrier
**Dietitian Support**							
10. Waiting for the dietitian to assess the patient.	**1**	**2**	**3**	**4**	**5**	**6**	**7**
11. Not enough dietitian time dedicated to the ICU during regular weekday hours.	**1**	**2**	**3**	**4**	**5**	**6**	**7**
12. No or not enough dietitian coverage during evenings, weekends and holidays.	**1**	**2**	**3**	**4**	**5**	**6**	**7**
13. There is not enough time dedicated to education and training on how to optimally feed patients.	**1**	**2**	**3**	**4**	**5**	**6**	**7**
**Delivery of Enteral Nutrition to the Patient**							
14. No feeding tube in place to start feeding.	**1**	**2**	**3**	**4**	**5**	**6**	**7**
15. Delay in physicians ordering the initiation of EN.	**1**	**2**	**3**	**4**	**5**	**6**	**7**
16. Waiting for physician/radiology to read x-ray and confirm tube placement.	**1**	**2**	**3**	**4**	**5**	**6**	**7**
17. Delays in initiating motility agents in patients not tolerating enteral nutrition (i.e. high gastric residual volumes).	**1**	**2**	**3**	**4**	**5**	**6**	**7**
18. Delays and difficulties in obtaining small bowel access in patients not tolerating enteral nutrition (i.e. high gastric residual volumes).	**1**	**2**	**3**	**4**	**5**	**6**	**7**
19. In resuscitated, hemodynamically stable patients, other aspects of patient care still take priority over nutrition.	**1**	**2**	**3**	**4**	**5**	**6**	**7**
20. Poor communication amongst the ICU team regarding the nutrition management resulting in delays in initiating or progression of EN.	**1**	**2**	**3**	**4**	**5**	**6**	**7**

Critical Care Provider Attitudes and Behaviour	Not at all Important	Unimportant	Somewhat Unimportant	Neither Important or Unimportant	Somewhat Important	Important	Very Important
	Not a Barrier →						→ Major Barrier
21. Non-ICU physicians (i.e. surgeons, gastroenterologists) requesting patients not be fed enterally.	**1**	**2**	**3**	**4**	**5**	**6**	**7**
22. Nurses failing to progress feeds as per the feeding protocol.	**1**	**2**	**3**	**4**	**5**	**6**	**7**
23. Feeds being held due to diarrhea.	**1**	**2**	**3**	**4**	**5**	**6**	**7**
24. Fear of adverse events due to aggressively feeding patients.	**1**	**2**	**3**	**4**	**5**	**6**	**7**
25. Feeding being held too far in advance of procedures or operating room visits.	**1**	**2**	**3**	**4**	**5**	**6**	**7**
26. General belief among ICU team that provision of adequate nutrition does not impact on patient outcome.	**1**	**2**	**3**	**4**	**5**	**6**	**7**

27. Reflecting on the 26 barriers to providing enteral nutrition listed above, are there any other barriers that hinder your ability to deliver adequate amounts of enteral nutrition?

____
____
____
____
____

28. Reflecting on the 26 barriers to providing enteral nutrition listed above, enter the number corresponding to the items that you believe are the 3 most important barriers to the provision of adequate EN in your ICU:

- First most important barrier: ☐
- Second most important barrier: ☐
- Third most important barrier: ☐

Part B: Personal Characteristics
Please fill in the circle that best corresponds to you.

1. What is your sex? Male o Female o

2. What is your age?
   20-34 years o
   35-49 years o
   50-64 years o
   65 years or older o

3. What is your primary clinical specialty?
   Dietitian o
   Nurse o
   Physician o Please specify:
      Intensivist (Medical) o
      Intensivist (Surgical) o
      Anaesthesia o
      Emergency Medicine o
      Internal Medicine o
      Surgeon o
      Other o
   Other clinical specialty o Please specify ___________________

4. In my clinical work I am dedicated to the ICU…..
   Full-time o
   Part-time o Full-time equivalent:________________
   Locum o
   Casual o
   Other o Please specify ___________________

5. How long have you been working in the ICU?
   0 - 5 years o
   6 - 10 years o
   11 - 15 years o
   Greater than 15 years o

6. Do you play a leadership role in the ICU? Yes o No o
   If yes, please specify:
   Medical Director o
   Nurse manager o
   Clinical Nurse Specialist o
   Charge Nurse o
   Other o

Additional Comments

You have now completed the questionnaire – thank you! In the space below, please make any additional comments you wish to make about barriers or solutions to providing adequate enteral nutrition to patients in your ICU.

Thank you very much for taking the time to complete this questionnaire.
Your contribution is valued.
